# Spontaneous biliary peritonitis with common bile duct stones: report of a case

**DOI:** 10.1186/s40792-016-0234-6

**Published:** 2016-09-27

**Authors:** Ryoga Hamura, Koichiro Haruki, Jun Tsutsumi, Sumio Takayama, Hiroaki Shiba, Katsuhiko Yanaga

**Affiliations:** 1 Department of Surgery, Mashiko Hospital, Saitama, Japan; 2 Department of Surgery, The Jikei University School of Medicine, 3-25-8, Nishi-Shinbashi, Minato-ku, Tokyo, 105-8461 Japan

**Keywords:** Spontaneous biliary peritonitis, Common bile duct stones

## Abstract

Spontaneous biliary peritonitis is rare in adults. We herein report a case of spontaneous biliary peritonitis. An 84-year-old man was admitted to our hospital for abdominal pain for 5 days. He developed fever, jaundice, and abdominal rigidity. Computed tomography (CT) revealed massive ascites in the omental bursa and around the liver. The ascites obtained by diagnostic paracentesis was dark yellow-green in color, which implied bile leakage. With a diagnosis of bile peritonitis, the patient underwent emergency exploratory laparotomy. There was massive biliary ascites in the abdominal cavity, especially in the omental bursa. Because exploration failed to demonstrate the perforation site in the gallbladder and biliary duct, we performed abdominal lavage alone. Postoperative endoscopic retrograde cholangiopancreatography showed stones in the common bile duct, and there was no evidence of biliary leakage. Endoscopic retrograde biliary drainage was performed using a plastic stent to reduce the pressure of the common bile duct. After the operation, the patient showed satisfactory recovery and started oral intake on postoperative day 8. However, the patient developed heart failure due to renal dysfunction with nephrotic syndrome at 1 month after the operation. With a diagnosis of rapidly progressive glomerulonephritis due to immune complex, the patient received steroid treatment for nephritis, diuretics, and carperitide for heart failure. Although heart failure and renal dysfunction improved by these treatment, the patients developed toxic epidermal necrolysis which was refractory to intensive treatments including steroid pulse and immunoglobulin, and the patient died 76 days after the operation.

## Background

Spontaneous biliary peritonitis is rare in adults. Kent et al. reported spontaneous biliary peritonitis caused by no detectable perforation of the gallbladder as “biliary peritonitis without perforation of the gallbladder” in 1974 [1]. The diagnosis of spontaneous biliary peritonitis is often delayed due to their nonspecific symptoms, which results in high morbidity. Early diagnosis of spontaneous biliary peritonitis and surgical intervention are of crucial importance [2–6]. We herein report a case of spontaneous biliary peritonitis.

## Case presentation

**Fig. 1:**
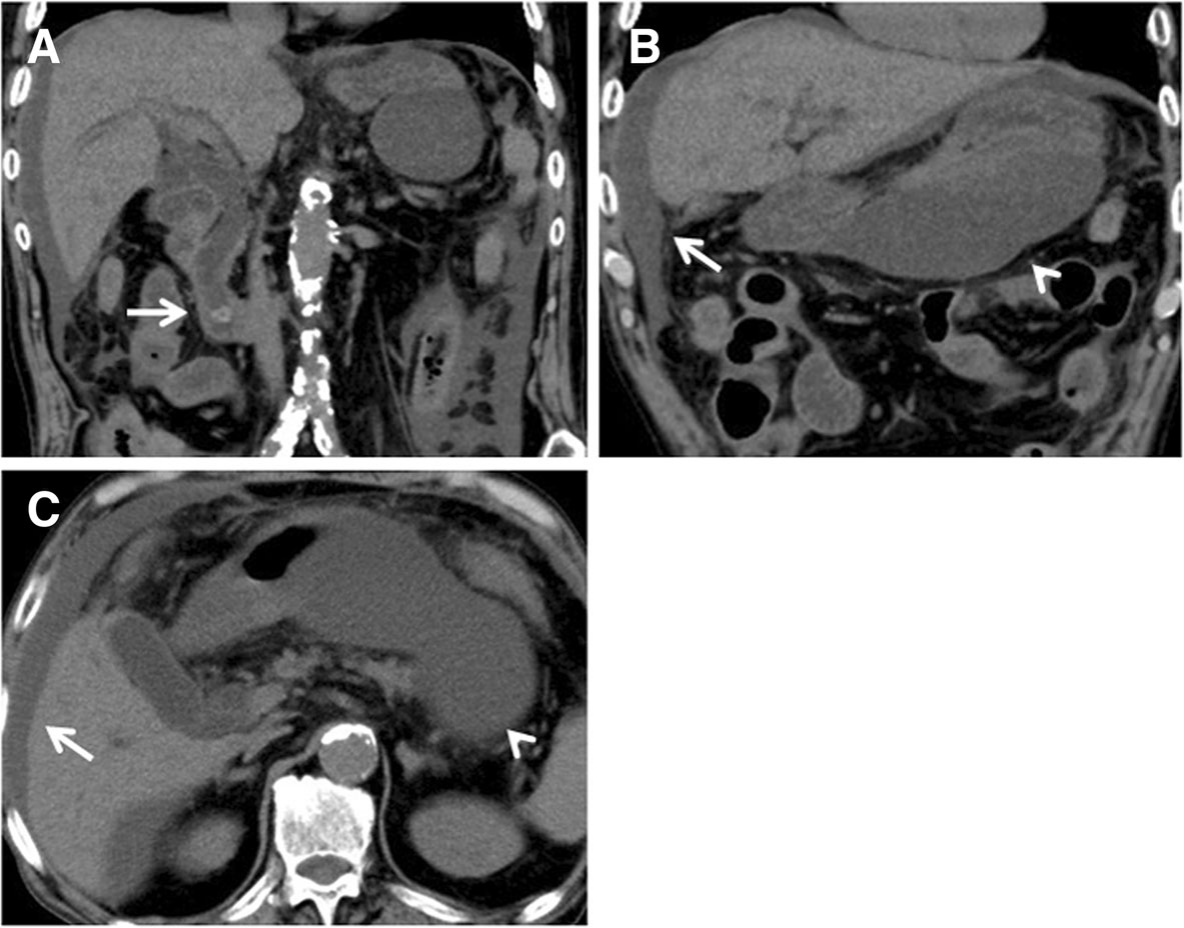
Computed tomography revealed common bile duct stones (**a**, *arrow*) and massive ascites in the omental bursa (**b**, **c**, *arrowhead*) and around the liver (**b**, **c**, *arrow*)

**Fig. 2:**
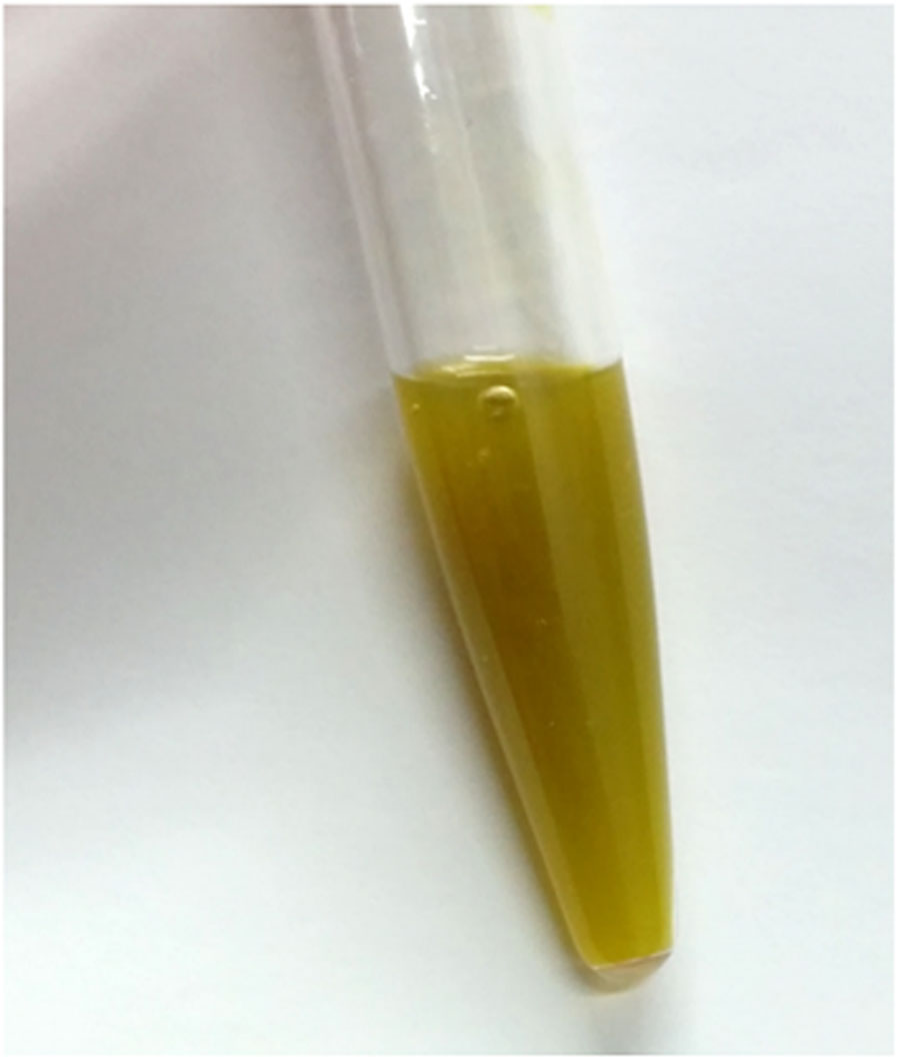
Diagnostic paracentesis demonstrated ascites with a dark yellow-green color

**Fig. 3:**
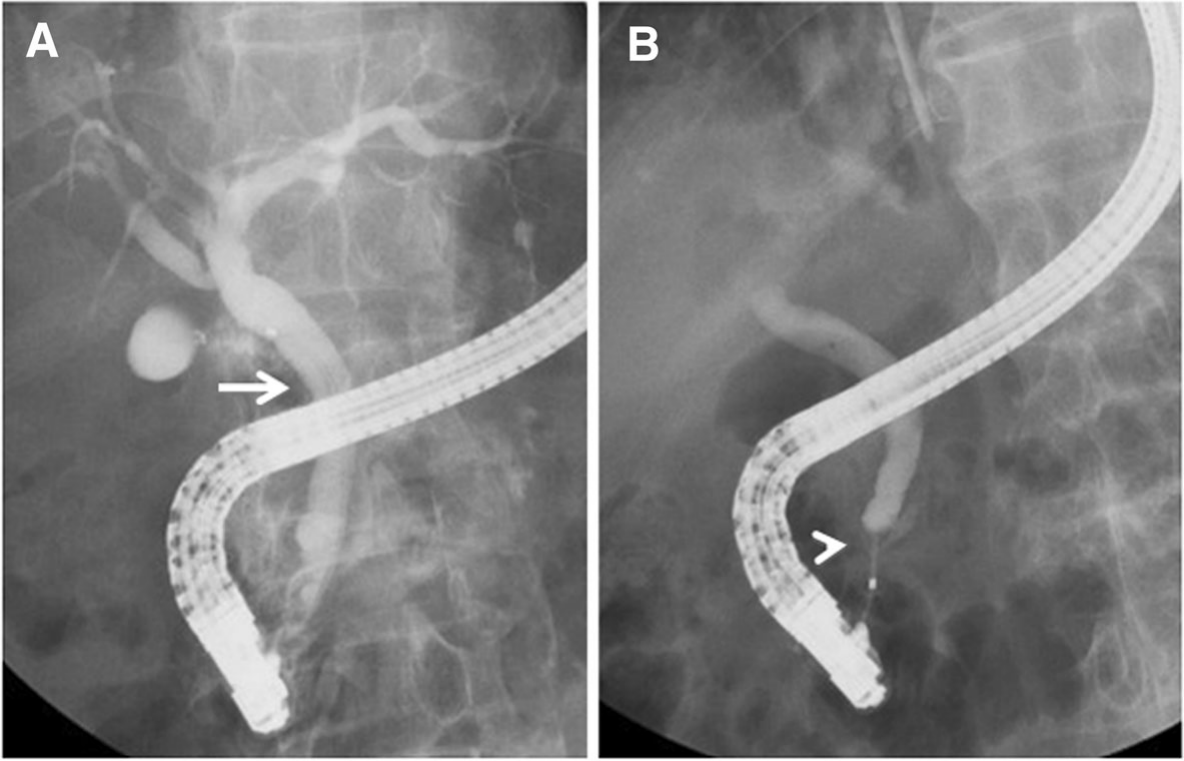
Postoperative endoscopic retrograde cholangiopancreatography (ERCP) showing several filling defects which are considered as gallstones in the common bile duct. There was no evidence of biliary leakage (**a**, *arrow*) and pancreaticobiliary maljunction (**b**, *arrowhead*)

An 84-year-old man, who had diabetes mellitus treated with insulin injection, was admitted to our hospital for abdominal pain for 5 days. He developed fever, jaundice, and abdominal rigidity. Laboratory data showed increased white blood cells of 15.3 × 10^3^/μl, serum C-reactive protein of 21.0 mg/dl, total bilirubin of 3.4 mg/dl, direct bilirubin of 2.4 mg/dl, aspartate aminotransferase of 65 IU/l, alanine aminotransferase of 45 U/l, alkaline phosphatase of 670 IU/l, gamma glutamyl transferase of 82 IU/l, and amylase of 359 U/l. Computed tomography (CT) revealed massive ascites in the omental bursa and around the liver (Fig. 1). The ascites obtained by diagnostic paracentesis demonstrated ascites with a dark yellow-green color, which implied bile leakage (Fig. 2). With a diagnosis of bile peritonitis, the patient underwent emergency laparotomy. There was massive biliary ascites in the abdominal cavity, especially in the omental bursa as shown by CT. Because exploration failed to demonstrate perforation site in the gallbladder and biliary duct, we performed abdominal lavage alone. Laboratory data of the ascites revealed total bilirubin of 3.4 mg/dl, and direct bilirubin of 2.4 mg/dl. *Klebsiella pneumoniae* was identified from the culture of the ascites. Postoperative endoscopic retrograde cholangiopancreatography (ERCP) showed several filling defects which are considered as gallstones in the common bile duct, and there was no evidence of biliary leakage and pancreaticobiliary maljunction (Fig. 3). Endoscopic retrograde biliary drainage (ERBD) was performed using a plastic stent to reduce the pressure of the common bile duct. After the operation, the patient showed satisfactory recovery and started oral intake on postoperative day 8. For reduced activities of daily living, the patient started rehabilitation, but at 1 month after the operation, the patient developed heart failure due to renal dysfunction with nephrotic syndrome. With a diagnosis of rapidly progressive glomerulonephritis due to immune complex, the patient received steroid treatment for nephritis, diuretics, and carperitide for heart failure. Although heart failure and renal dysfunction improved by these such treatments, the patient developed skin eruption on his face and body trunk 2 months after the operation. With a diagnosis of drug eruption, the suspected drugs were discontinued, but the skin eruption rapidly expanded with vesicles and fell into the status of toxic epidermal necrolysis. Despite intensive treatments including steroid pulse and immunoglobulin, the patient died 76 days after the operation. A drug-induced lymphocyte stimulation test revealed that the drug eruption was caused by trimethoprim-sulfamethoxazole.

### Discussion

Biliary peritonitis is caused by perforation of the gallbladder, bile duct, or upper gastrointestinal tract [4, 5]. Nontraumatic perforation of the bile duct is a disease in which the extrahepatic duct or intrahepatic duct is perforated spontaneously without traumatic or iatrogenic injury [7]. It has also been described as spontaneous perforation of the bile duct [3, 6–10]. Although the most frequent cause of bile duct injury is iatrogenic, it sometimes occurs after invasive procedures such as ERCP and cholecystectomy [7, 8, 10]. Spontaneous perforation of the extrahepatic bile duct is rare. The pathogenesis of nontraumatic perforation of the bile duct has not been established and may be related to a single or multiple factors. It has been suggested that any disease increasing the intraductal pressure, stasis, distal obstruction of the bile duct, diverticulum or abnormal glands in the bile duct wall, infection of bile duct, or ischemia may result in perforation of the duct wall. Sudden increase in pressure by such as biliary stones is associated with bile duct perforation [8]. Spontaneous perforation of gallbladder has been reported to be associated infections, malignancies, stones, thrombosis of intramural vessels, increased biliary pressures, use of corticosteroids and systemic diseases such as diabetes mellitus and atherosclerotic heart disease [3, 5, 9, 11]. In the present case, the perforation site was not detected in the biliary tree during the operation and by ERCP. The possible reason was that five days had passed since the onset to the operation. The patient had common bile duct stones and diabetes mellitus as risk factors and the fluid collection accumulated mainly in the omental bursa. From these points, the perforation site was suspected to be the extrahepatic duct and possible mechanisms in this case were that impaction of common bile duct stones might cause erosion and weakness of the bile duct wall and increased intraductal pressure resulted in the perforation. If intraoperative cholangiography was performed, it might have helped to detect the perforation site. However, the patients could not endure the additional procedure during the operation.

Spontaneous biliary peritonitis is difficult to diagnose before operation. The clinical features of nontraumatic perforation of the bile duct are nonspecific and similar to gallbladder perforation. The delay in diagnosis is the major cause of its high morbidity and mortality [2–8, 11, 12]. In the literature, ultrasonography and CT were very informative for detecting the primary lesion and perihepatic fluid collection [8]. Moreover, paracentesis is helpful for the diagnosis of biliary peritonitis. In the present case, the distribution of the ascites on CT and the sample of the ascites helped to reach the diagnosis of biliary peritonitis.

Surgical intervention is an effective treatment for biliary peritonitis. It is important to drain the abdominal contamination caused by infected bilious peritoneal fluid [8, 9]. In most cases, cholecystectomy has been performed for biliary peritonitis with gallbladder perforation [4]. In the current case, the patient was treated with abdominal lavage and postoperative ERBD and achieved temporary remission. Abdominal lavage and postoperative ERBD were sufficient when no perforation site was detected.

## Conclusions

Early diagnosis and surgical intervention are important to improve outcomes of spontaneous biliary peritonitis. At least, abdominal lavage and biliary drainage including ERBD are needed for spontaneous biliary peritonitis, if no perforation site was identified.
